# Integrating miRNA and full-length transcriptome profiling to elucidate the mechanism of muscle growth in Muscovy ducks reveals key roles for miR-301a-3p/ANKRD1

**DOI:** 10.1186/s12864-024-10138-z

**Published:** 2024-04-04

**Authors:** Jiangnan Huang, Xiaolan Xiong, Weihong Zhang, Xiaolian Chen, Yue Wei, Haiqin Li, Jinfang Xie, Qipeng Wei, Quanyong Zhou

**Affiliations:** Institute of Animal Husbandry and Veterinary Medicine, Jiangxi Academy of Agricultural Sciences, Nanchang, 330200 China

**Keywords:** Muscovy ducks, Muscle growth, microRNA sequencing, Full-length transcriptome, miR-301a-3p, ANKRD1

## Abstract

**Background:**

The popularity of Muscovy ducks is attributed not only to their conformation traits but also to their slightly higher content of breast and leg meat, as well as their stronger-tasting meat compared to that of typical domestic ducks. However, there is a lack of comprehensive systematic research on the development of breast muscle in Muscovy ducks. In addition, since the number of skeletal muscle myofibers is established during the embryonic period, this study conducted a full-length transcriptome sequencing and microRNA sequencing of the breast muscle. Muscovy ducks at four developmental stages, namely Embryonic Day 21 (E21), Embryonic Day 27 (E27), Hatching Day (D0), and Post-hatching Day 7 (D7), were used to isolate total RNA for analysis.

**Results:**

A total of 68,161 genes and 472 mature microRNAs were identified. In order to uncover deeper insights into the regulation of mRNA by miRNAs, we conducted an integration of the differentially expressed miRNAs (known as DEMs) with the differentially expressed genes (referred to as DEGs) across various developmental stages. This integration allowed us to make predictions regarding the interactions between miRNAs and mRNA. Through this analysis, we identified a total of 274 DEGs that may serve as potential targets for the 68 DEMs. In the predicted miRNA‒mRNA interaction networks, let-7b, miR-133a-3p, miR-301a-3p, and miR-338-3p were the hub miRNAs. In addition, multiple DEMs also showed predicted target relationships with the DEGs associated with skeletal system development. These identified DEGs and DEMs as well as their predicted interaction networks involved in the regulation of energy homeostasis and muscle development were most likely to play critical roles in facilitating the embryo-to-hatchling transition. A candidate miRNA, miR-301a-3p, exhibited increased expression during the differentiation of satellite cells and was downregulated in the breast muscle tissues of Muscovy ducks at E21 compared to E27. A dual-luciferase reporter assay suggested that the ANKRD1 gene, which encodes a transcription factor, is a direct target of miR-301a-3p.

**Conclusions:**

miR-301a-3p suppressed the posttranscriptional activity of ANKRD1, which is an activator of satellite cell proliferation, as determined with gain- and loss-of-function experiments. miR-301a-3p functions as an inducer of myogenesis by targeting the ANKRD1 gene in Muscovy ducks. These results provide novel insights into the early developmental process of black Muscovy breast muscles and will improve understanding of the underlying molecular mechanisms.

**Supplementary Information:**

The online version contains supplementary material available at 10.1186/s12864-024-10138-z.

## Introduction

Skeletal muscle development plays a crucial role in duck production as it directly affects the quantity and quality of meat. This, in turn, has a significant impact on the overall economic value of ducks. The development of embryonic skeletal muscle is a key factor in both muscle growth rate and meat quality. This is because the number of skeletal muscle fibers is determined during the embryonic period, and postnatal muscle mass increase mainly occurs through the fusion of satellite cells to expand the fibers [1, 2]. Therefore, studying skeletal muscle development during the embryonic period is essential for guiding genetic improvement in ducks.

With the advancement of high-throughput sequencing technologies, transcriptome sequencing and small RNA sequencing have emerged as effective methods for identifying candidate genes and miRNAs related to muscle development. Comparative transcriptome analysis has revealed several evolutionarily conserved genes, including TNNI2, MYL4, IGF2BP1, CSRP3, and SPP1, that are associated with muscle development [3–6]. Increasing evidence suggests that miRNAs play a critical role in regulating skeletal muscle development. Gu et al. [7] and Li et al. [8] identified 382 and 1091 differentially expressed miRNAs during duck embryonic breast muscle development through miRNA sequencing. Additionally, previous studies have shown that miRNAs, including miRNA‐1, miRNA‐133, miRNA-33a, and miR-365, regulate target genes involved in myoblast proliferation and differentiation [9, 10].

In China, Muscovy ducks are popular due to their stronger-tasting meat compared to domestic ducks (Anas platyrhynchos). However, there is limited understanding of the molecular mechanisms involved in the maturation of skeletal muscle during the later postnatal period, especially regarding the control of genes, pathways, and miRNAs related to breast muscle development. Therefore, it is necessary to investigate miRNA and mRNA expression profiles to comprehend the molecular regulatory mechanism and identify key miRNA targets for breast muscle development in Muscovy ducks.

 However, RNA-seq does have some inherent shortcomings. Firstly, the fragments need to be broken and then spliced, which can result in incomplete fragments. Secondly, the quality of the generated transcripts is often low, potentially leading to inaccurate annotations. Lastly, due to limited reading space, the entire text cannot be read [11]. On the other hand, full-length PacBio SMRT Iso-seq offers several advantages. Firstly, it directly captures complete transcripts without the need for fragmentation and splicing [12]. Additionally, it provides longer reading time and more comprehensive content. Thus, Iso-seq serves to address the limitations of RNA-seq.

This study employed a combination of full-length transcriptome sequencing (PacBio and Illumina sequencing) and microRNA sequencing to analyze the miRNA-mRNA expression profiles in Fujian black Muscovy ducks during four stages of breast muscle development: two prenatal stages (21, 27 dpc) and two postnatal stages (0, 7 dpn). The integration of miRNA and mRNA expression profile data allowed the construction of a miRNA-mRNA regulatory network associated with Muscovy duck muscle growth and development. The differentially expressed miRNAs and target genes identified in this study will provide insights into the molecular mechanisms underlying Muscovy duck muscle development and serve as a basis for duck breeding.

## Results

### General properties of PacBio sequencing

**Fig. 1:**
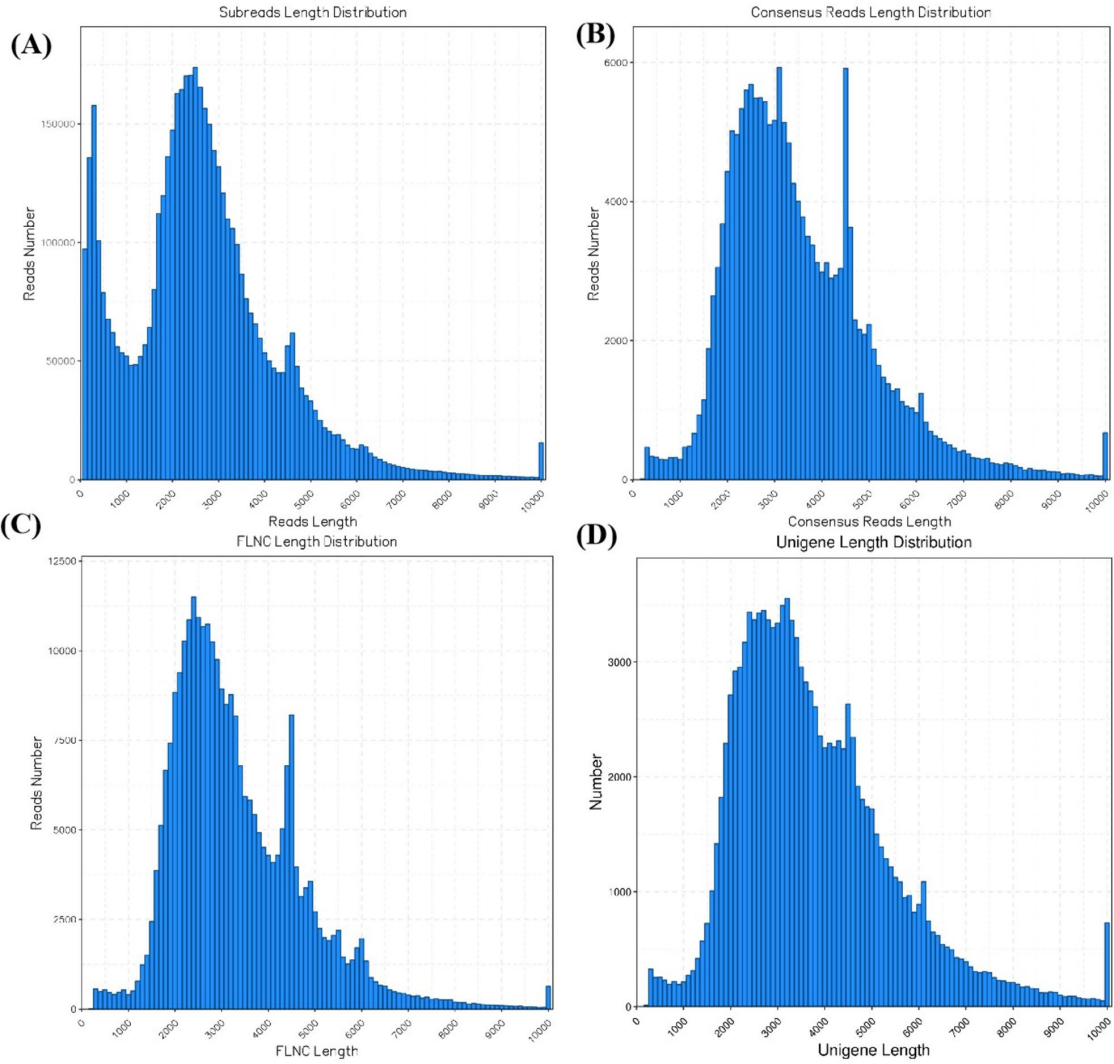
Length distributions of PacBio SMRT sequencing. **A** Number and length distributions of 4,982,603 Subreads sequences. **B** Number and length distributions of 347,932 CCS sequences. **C** Number and length distributions of 284,128 FLNC sequences. **D** Number and length distributions of 109,718 corrected sequences

To investigate the transcriptome in Muscovy ducks, we conducted a comprehensive analysis of six different tissues: the cerebrum, rumen, liver, spleen, renal cortex, and longissimus muscle. We utilized the Pacific Bioscience Sequel platform to sequence a pooled RNA sample, enabling us to accurately capture complete sequences and discover full-length splice variants. Through SMRT sequencing, we successfully obtained a total of 4,982,603 subreads (equivalent to 12.99 GB). These subreads had an average length of 2608 bp and an impressive N50 of 3229 bp. To ensure precise sequence information, we generated consensus circular sequences (CCSs) from subreads that had passed through the insert at least twice, resulting in 347,932 CCSs. Among them, 286,688 CCSs were complete reads and 284,128 were identified as FLNC (Full-Length Non-Chimeric) reads, indicating the minimal presence of artificially formed concatenations. The average length of FLNC reads was 3,275 bp. For more details on the length distribution of subreads, CCSs, and FLNC reads, please refer to Supplemental Table S5 and Fig. 1.

### Error correction of PacBio long reads using Illumina reads

To address the high error rates associated with PacBio long reads, we generated 7.87 GB of clean reads through NGS sequencing on clean data. These clean reads were then used to correct the consensus isoform sequences obtained from PacBio long reads. The correction was performed using LoRDEC software, resulting in 171,439 corrected sequences. These corrected sequences had an N50 length of 3,962 bp and a mean read length of 3,525 bp. We obtained a total of 109,718 nonredundant transcripts (Supplemental Table S5), which were further analyzed after being annotated as unigenes.

### Comparison of the results of SMRT sequencing and next-generation sequencing

The majority of contigs obtained from Illumina sequencing ranged from 200 to 500 bp in length, with only a small percentage exceeding 2 kb. Similarly, a significant proportion of unigenes also fell within the 200–500 bp range. In contrast, SMRT sequencing resulted in a larger number of longer transcripts, with a combined length of 414,495,772 bp. On the other hand, Illumina sequencing produced a higher total number of contigs and unigenes, amounting to 499,432,484 bp (Supplemental Table S6 and Supplemental Table S7).

### Functional annotation of transcripts

**Fig. 2:**
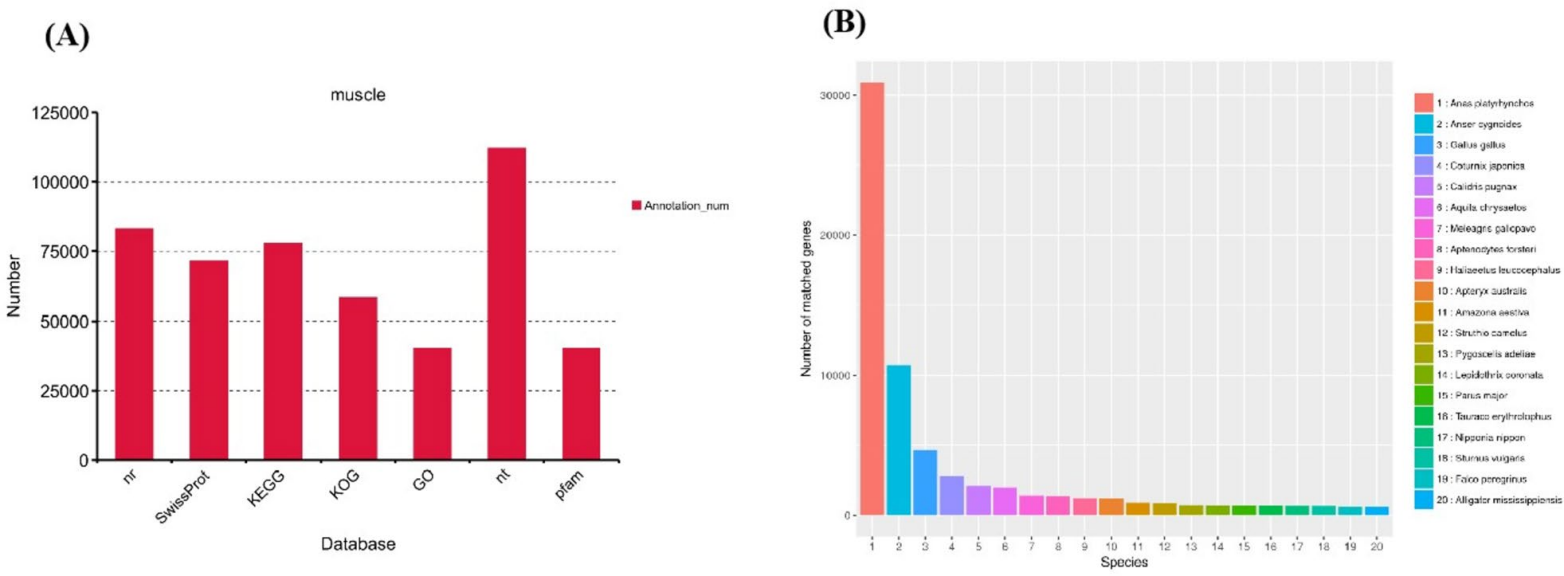
Function annotation of novel genes. **A** Function annotation of novel genes in all databases (NR, NT, Pfam, KOG, Swiss-Prot, KEGG, and GO). **B** Nr Homologous species distribution diagram of novel genes

By searching against several databases, a significant number of transcripts were successfully annotated. The NR database had the highest proportion of annotations, with 69.51% of transcripts aligned to Anas platyrhynchos. Anser cygnoides was the second most aligned species (Fig. 2 and Supplemental Table S8).

### Differentially expressed genes and miRNAs in Muscovy duck breast muscle at different stages

A total of 2286 DEGs were identified in the comparison between E21 and E27, E27 and D0, D0 and D7 with the highest number observed in the comparison between E21 and E27 stages. The miRNA sequencing results revealed 70 differentially expressed miRNAs (DEMs) in the three pairwise comparisons. Out of these, 55 were identified as known miRNAs, while 15 were classified as novel. The most prevalent category of small RNAs had a length of 22 nt (Supplemental Table S9 and Supplemental Table S10).

### Integrated analysis of DEGs and DEMs

In order to investigate the biological functions of differentially expressed miRNAs in breast muscle development, we conducted an analysis to predict the potential target genes of these miRNAs through three distinct comparisons. The miRanda software was used for this purpose. To explore the interactions between miRNAs and mRNA, we integrated the differentially expressed miRNAs (DEMs) with the differentially expressed genes (DEGs) identified during various developmental stages. As a result, we compiled a list of DEGs that could potentially be targeted by the DEMs. This comprehensive list can be found in Supplemental Table S6 and Supplemental Table S11. Subsequently, we focused on the associations between the miRNAs and target genes, specifically examining the miRNA‒mRNA pairs with negative associations, which were considered to be biologically significant.

**Fig. 3:**
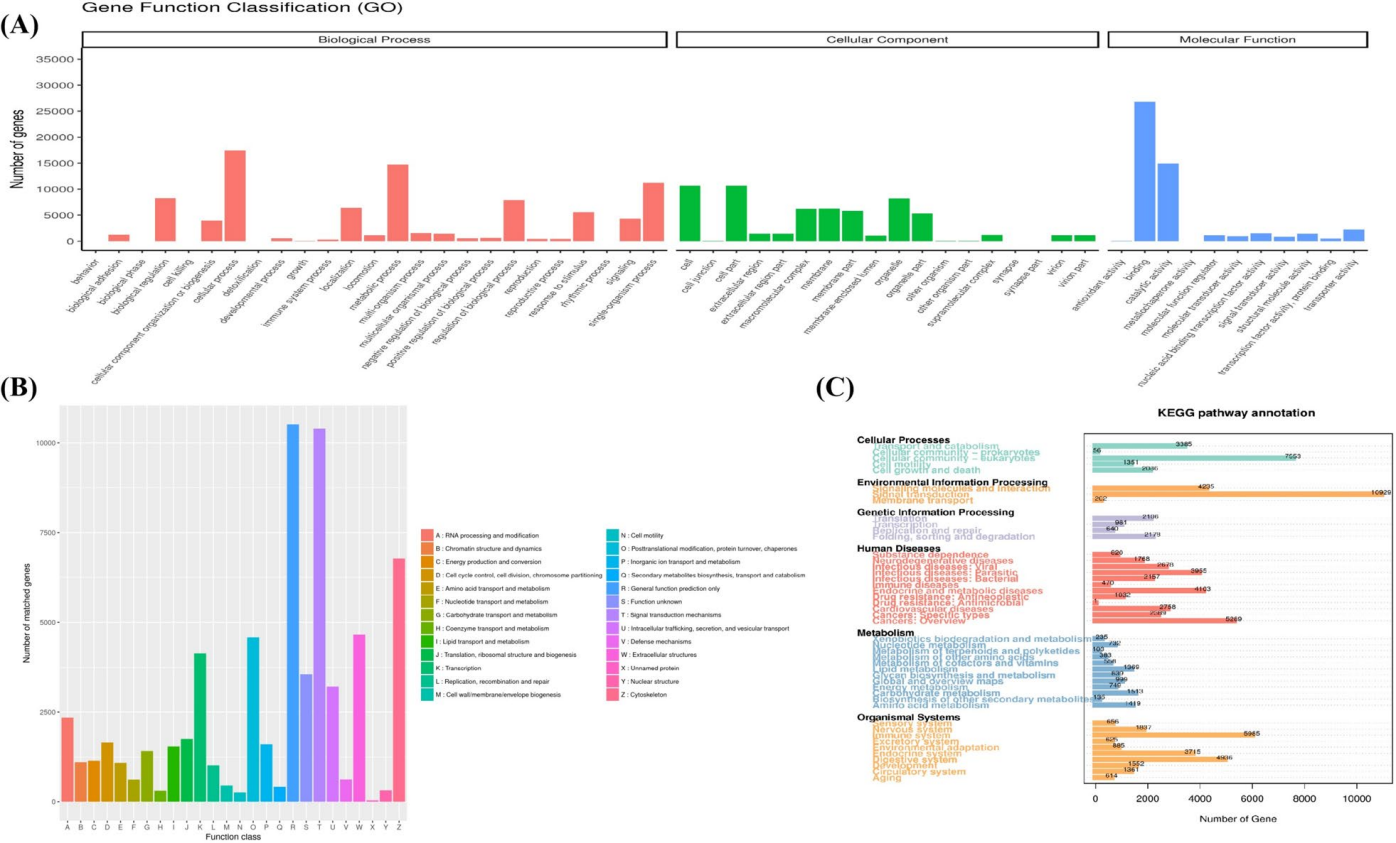
**A** Distribution of GO terms for all annotated transcripts. Red represents biological process; green represents cellular component; and blue represents molecular function. Te x-axis represents GO categories; the y-axis (right) represents the number of transcripts; (**B**) KOG enrichment of novel genes. **C** KEGG pathways enrichment of novel genes

To further explore the functional aspects of miRNA‒mRNA pairs with negatively correlated expression patterns during developmental stages, we conducted GO enrichment analysis for each comparison. The purpose of this analysis was to identify enriched biological process terms (*P* < 0.05). Our investigation revealed that the DEGs were actively involved in multiple processes, including: muscle tissue morphogenesis, the transition between fast and slow fibers, creatine metabolism, muscle cell differentiation, fatty acid metabolism, gluconeogenesis, energy homeostasis, and myoblast fusion (Fig. 3A and Supplemental Table S12). KOG analysis showed the novel genes were assigned to 26 functional clusters, and the “general function prediction only,” “signal transduction mechanisms,” and “cytoskeleton” ranked as the top three largest categories (Fig. 3B). Notably, The KEGG results demonstrated that the DEGs were mainly associated with key pathways such as endocytosis, cGMP-PKG signaling, insulin resistance, calcium signaling, PI3K-Akt signaling, and protein digestion and absorption. Detailed information on the top 10 KEGG terms from these six sets can be found in Fig. 3C and Table S13.

### Interaction network of differentially expressed genes and microRNAs

**Fig. 4:**
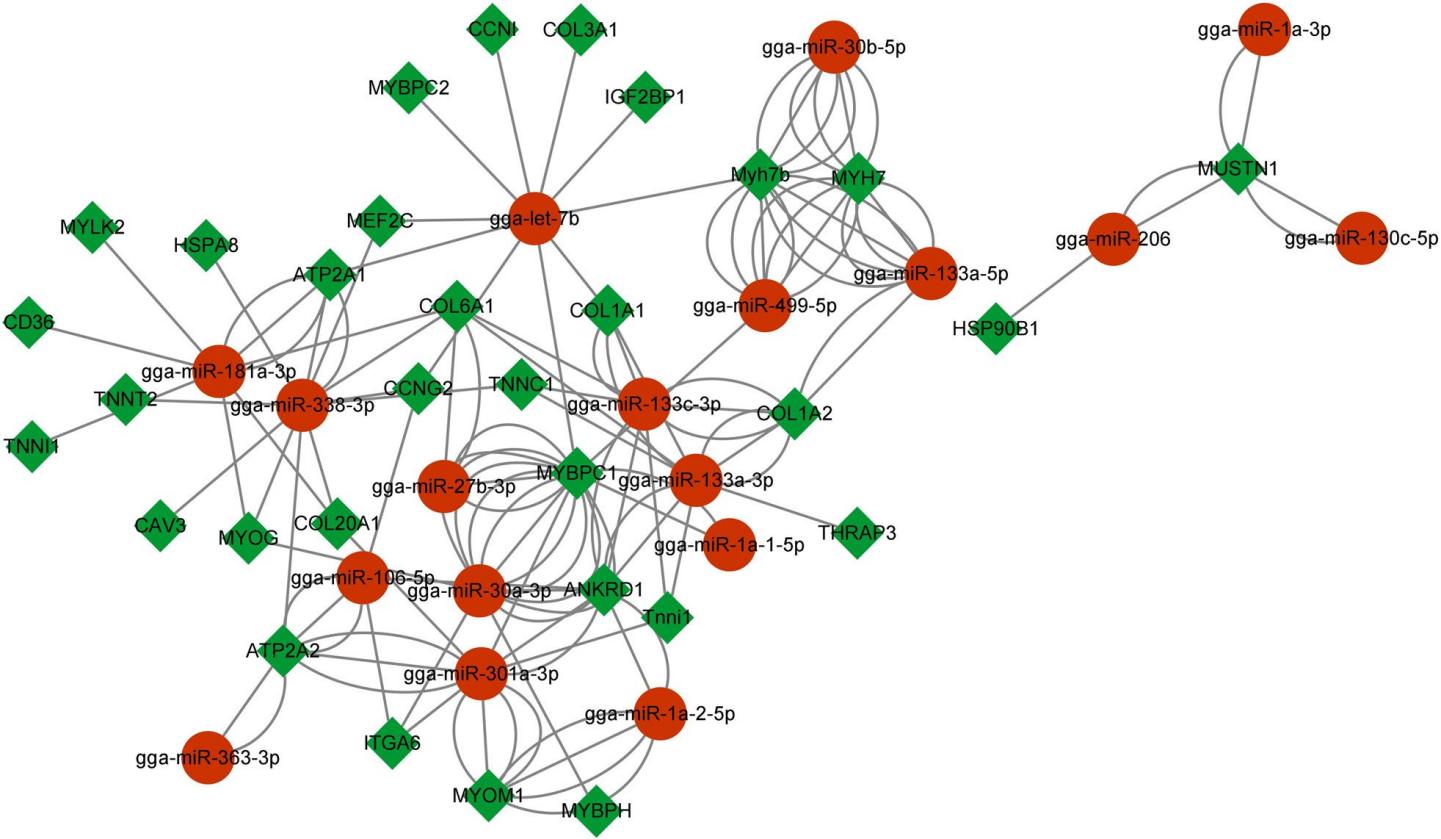
Interaction pattern of DEGs and DEMs. By taking the intersections of DEGs and target genes of differently expressed miRNAs (DEMs), we constructed an interaction network between differently expressed genes (DEGs) and DEMs with Cytoscape. Red and green nodes represent DEMs and differently expressed genes (DEGs), respectively

The objective of this study was to demonstrate the potential negative interactions between differentially expressed miRNAs and mRNAs in Muscovy duck breast muscle, which may contribute to variations in muscle development and adipose deposition. To achieve this, regulatory networks of miRNA‒mRNA pairs were constructed (Fig. 4). The study identified the core microRNAs and genes. Among the 18 microRNAs with significant regulatory functions in the network (e.g., gga-let-7b, gga-miR-133a-3p, gga-miR-1a-1-5p, gga-miR-206, and gga-miR-301a-3p), most of the genes (e.g., ANKRD1, ATP2A1, IGF2BP1, MEF2C, and MUSTN1) were regulated by at least two microRNAs. Notably, MEF2C and ANKRD1 were implicated in skeletal muscle cell differentiation, MYOG and CAV3 in myotube differentiation regulation, and COL1A1 and COL1A2 in skeletal system development.

### Validation of sequencing data by qRT‒PCR

**Fig. 5:**
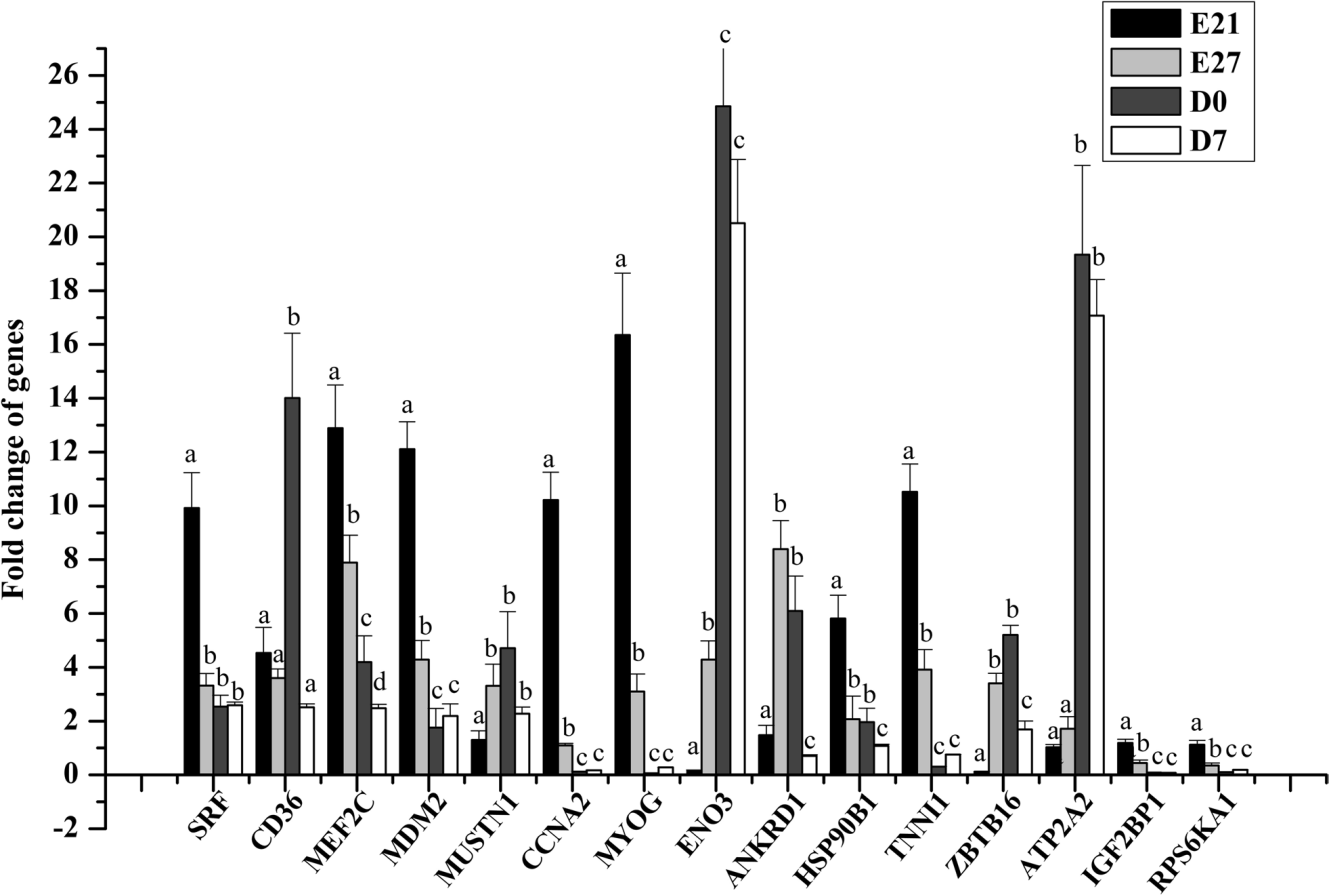
Quantitative RT‒PCR (qRT‒PCR) validation of DEGs at different developmental stages (E21, E27, D0, and D7). The x-axis represents the sample name, and the y-axis represents the relative expression level (2-dd-Ct). Data are expressed as mean ± SD of three biological replicates. Different small letters above the bars indicate significant difference among the different sample groups (*p* < 0.05)

**Fig. 6:**
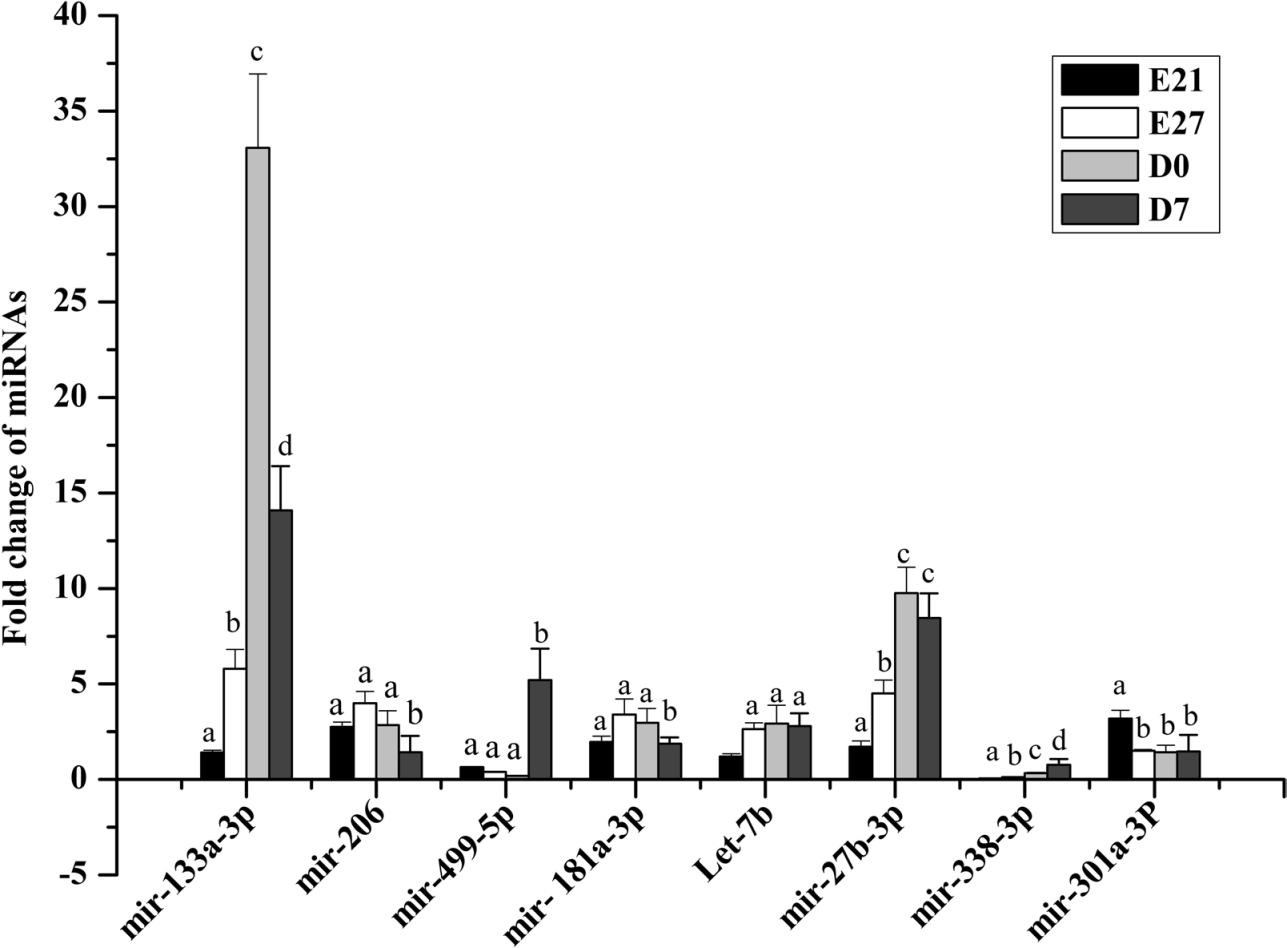
Quantitative RT‒PCR (qRT‒PCR) validation of DEMs at different developmental stages (E21, E27, D0, and D7). The x-axis represents the sample name, and the y-axis represents the relative expression level (2^−dd−Ct^). Data are expressed as mean ± SD of three biological replicates. Different small letters above the bars indicate significant difference among the different sample groups (*p* < 0.05)

The objective of this research was to validate the transcriptome and microRNA sequencing data using quantitative real-time PCR (qRT-PCR) analysis. A total of 10 miRNAs and 15 genes were comprehensively examined with qRT-PCR. The results from qRT-PCR were consistent with the sequencing data, providing mutual validation between the two techniques (see Figs. 5 and 6).

### Expression of miR-301a-3p and ANKRD1 during satellite cell differentiation

**Fig. 7:**
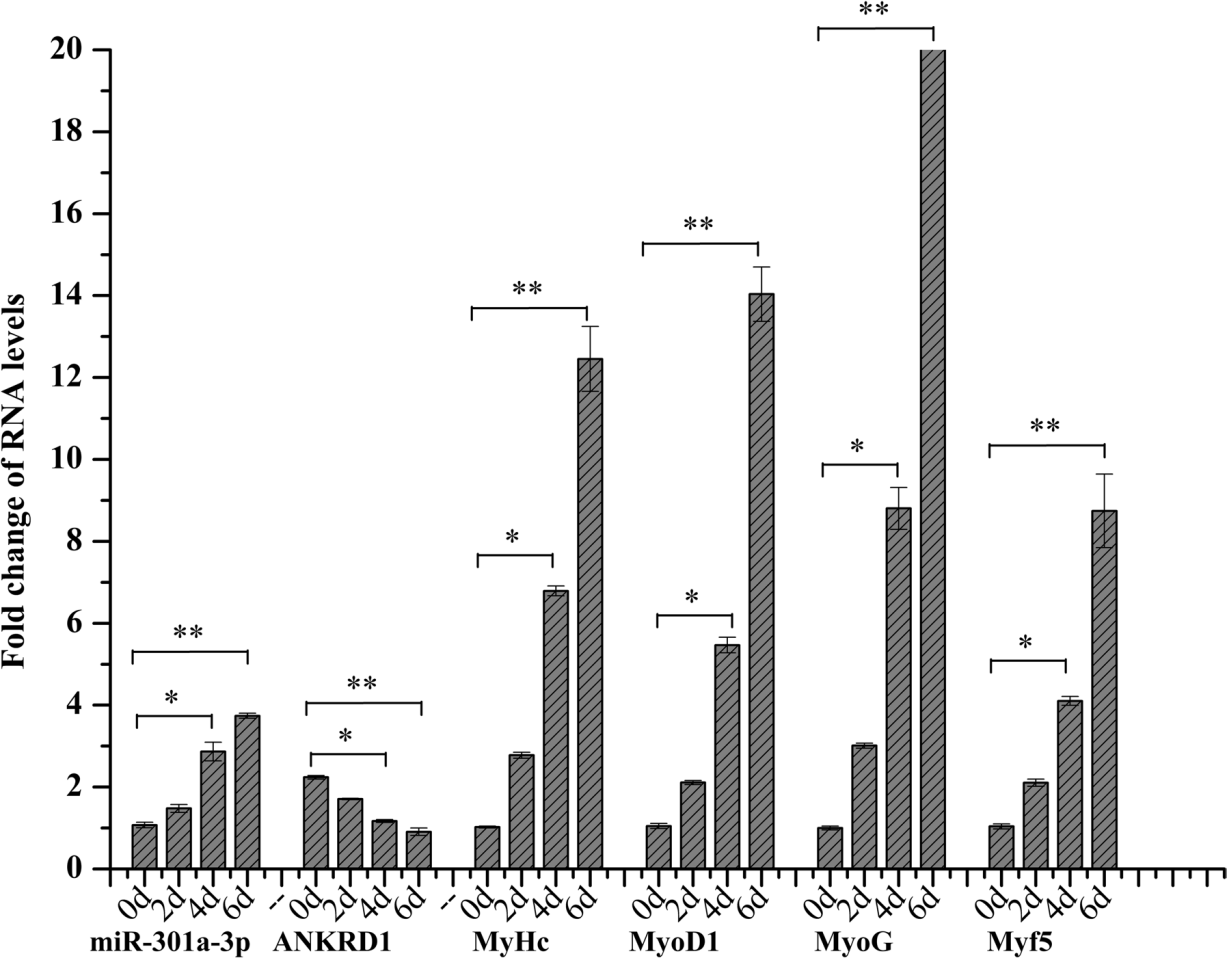
Expression patterns of miR-301a-3p, ANKRD1 and Myogenic differentiation marker gene during satellite cell differentiation of Muscovy duck. Primary myosatellite cells were treated with myogenic differentiation medium (2% horse serum, 1% double antibodies, 1% GlutaMAX, 90% high-glucose DMEM) for 0, 2, 4, and 6 days, and the expression levels of miR301a-3p, ANKRD1 and the myoblast differentiation genes MyHC, MyoD, MyoG and Myf5. The data are expressed as the mean ± SD (*n* = 3). *P < 0.05 and **P < 0.01 for comparisons between groups

The present study investigated the expression levels of miR-301a-3p during satellite cell (SC) differentiation. The results presented in Fig. 7 demonstrated a gradual increase in miR-301a-3p levels as SC myogenic development progressed, while the expression of the ANKRD1 gene decreased simultaneously. Additionally, the expression pattern of miR-301a-3p closely resembled that of key myogenic regulatory factors such as MyoG, Myod1, Myf5, and MyHC during SC myogenic differentiation (Fig. 7). These findings suggest that miR-301a-3p and ANKRD1 may play a crucial role in the process of myogenic differentiation.

### miR-301a-3p promotes satellite cell proliferation

**Fig. 8:**
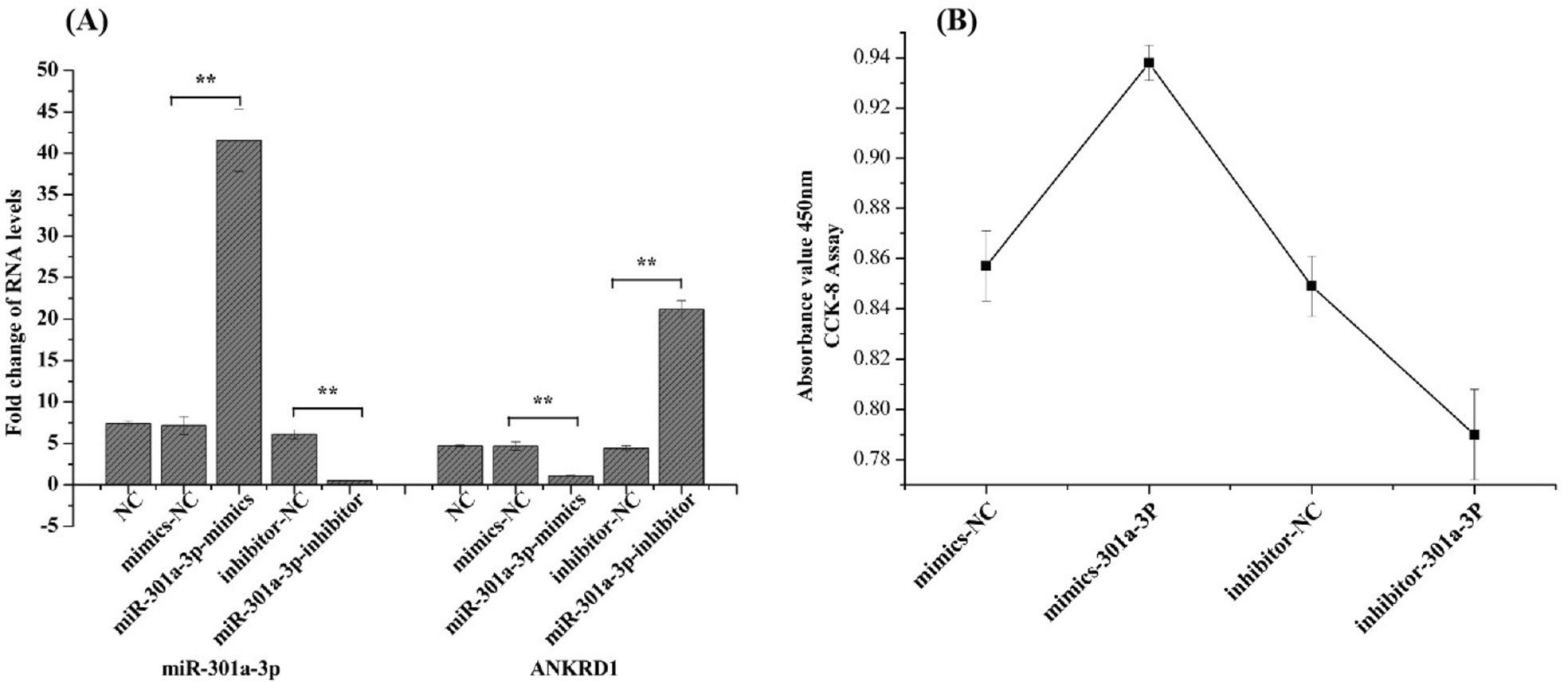
**A** Expression levels of miR-301a-3p and ANKRD1 transfected with miR-301a-3p mimics, miR-301a-3p inhibitor, negative control (NC) and mimics negative control (NC) at 48 h post-transfection; (**B**) CCK-8 assay of Muscovy duck muscle satellite cells transfected with miR-301a-3p agomir and miR-301a-3p agomir negative control (NC) at 48 h post-transfection; The data are expressed as the mean ± SD (*n* = 3). *P < 0.05 and **P < 0.01 for comparisons between groups

To investigate the potential role of miR-301a-3p in satellite cell (SC) proliferation, SCs cultured in GM were transfected with either miR-301a-3p mimic or miR-301a-3p inhibitor. Our findings indicate that the expression of miR-301a-3p was significantly upregulated (*P* < 0.01) by the miR-301a-3p mimic and downregulated (*P* < 0.01) by the miR-301a-3p inhibitor (Fig. 8A). The results of the CCK-8 assay revealed that overexpression of miR-301a-3p at 24 h significantly increased the number of viable cells compared to the treatment with the negative control (NC) or miR-301a-3p inhibitor (Fig. 8B).

### The ANKRD1 gene is a direct target of miR-301a-3p

**Fig. 9:**
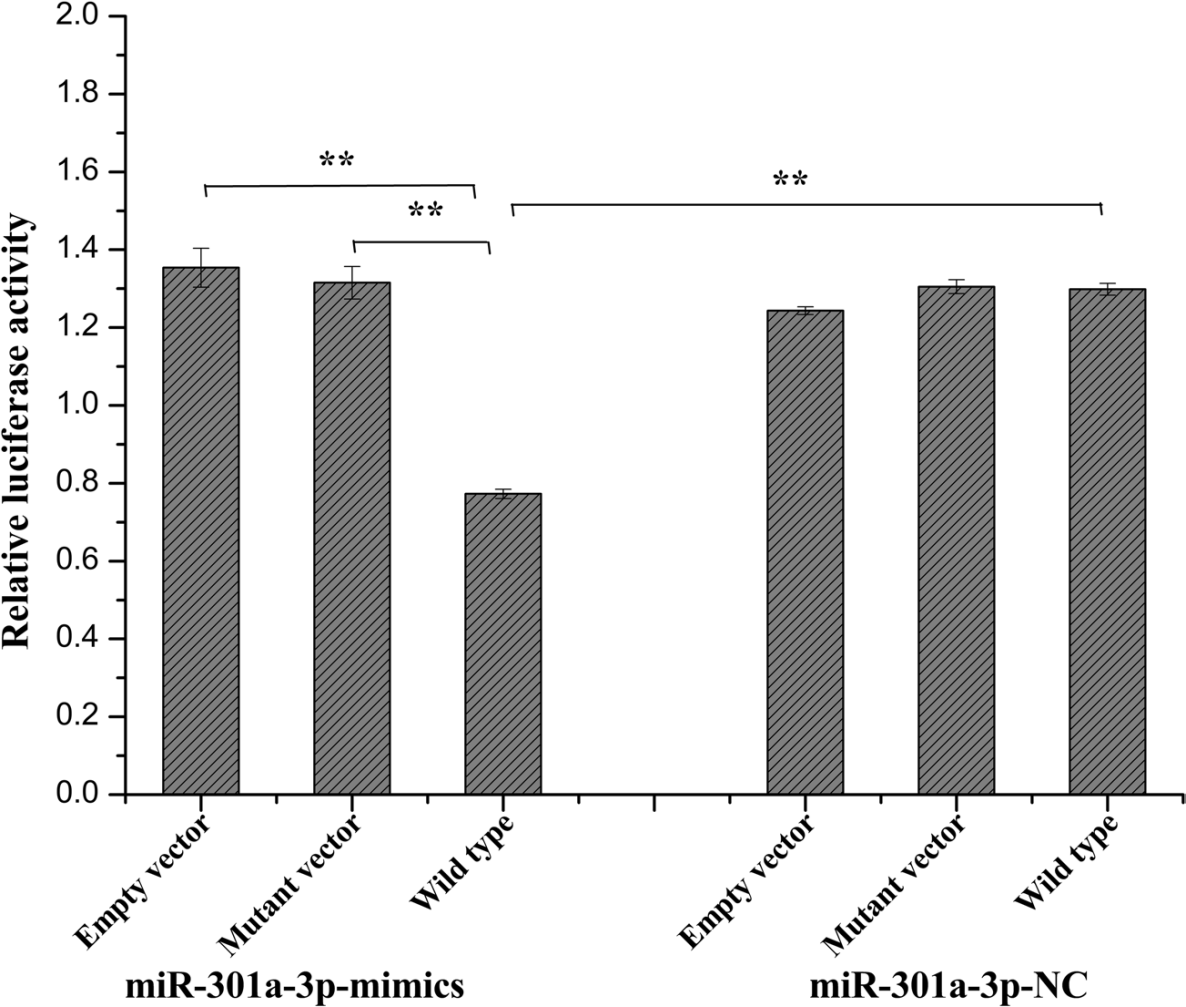
Validation of the interaction between miR-301a-3p and the 3′ UTR of the ANKRD1 gene via a dual-luciferase reporter assay in satellite cells. The data are presented as the mean ± SD (*n* = 3). *P < 0.05 and **P < 0.01 for comparisons between groups

In our study, we observed that miR-301a-3p was down-regulated from E21 to E27, while ANKRD1 was up-regulated during the same period (Figs. 5 and 6). Furthermore, our findings indicate that miR-301a-3p has the ability to bind to the 3′ UTR of the ANKRD1 gene. To confirm this binding relationship, we conducted a luciferase analysis using a dual-luciferase reporter system. Our results show that when mir-301a-3p mimic and pmirGLO-ANKRD1-3′-UTR were cotransfected into Muscovy duck skeletal muscle satellite cells, luciferase activity was significantly suppressed (Fig. 9). However, cotransfection of mir-301a-3p mimic and pmirGLO-ANKRD1-3′-UTR-mut did not have a significant effect on luciferase activity (Fig. 9). Furthermore, qRT-PCR analysis revealed a significant decrease in ANKRD1 mRNA expression levels after transfection with mir-301a-3p mimic in skeletal muscle satellite cells. Conversely, inhibiting mir-301a-3p led to an increase in ANKRD1 mRNA expression in these cells, as shown in Fig. 8A.

## Discussion

Sequencing a large volume of cDNA is essential for reconstructing and annotating the transcriptome. However, limitations in sequencing technologies often require a trade-off between sampling depth and completeness [13]. Additionally, gene structure characterization is prone to inaccuracies introduced by biosoftware and algorithms during the assembly of RNA-Seq short reads [11]. In this study, we employed PacBio single-molecule long-read sequencing (SMRT) technology in conjunction with NGS sequencing to determine the comprehensive transcriptomic landscape of Muscovy ducks. Our dataset provides the first SMRT sequencing atlas of the entire Muscovy duck transcriptome. We performed numerous annotation analyses using 109,718 high-quality transcripts. NR annotation revealed that 25.52% of the sequences aligned with Anas platyrhynchos sequences, while 9.11% aligned with Anser cygnoides sequences. These findings suggest that Muscovy ducks are most closely related to Anas platyrhynchos in terms of protein alignment, based on the current NR database.

Muscle fibre numbers primarily increase during embryonic periods, and these numbers significantly impact the postnatal accretion of muscle mass [14]. Therefore, it is crucial to comprehend muscle development during the embryonic stages. In this study, researchers utilized RNA-Seq to generate cDNA libraries for four developmental stages of the Muscovy duck in order to investigate the mechanisms of skeletal muscle development. By conducting pairwise comparisons, a total of 1455 differentially expressed genes (DEGs) were identified in the breast muscle libraries at different developmental stages. The study revealed that regional differences in gene expression were more pronounced during the earlier stages of embryonic development compared to the late postnatal stage. To validate the results obtained through RNA-Seq, the researchers confirmed 15 DEGs involved in muscle development using qRT-PCR. The consistency between the results from both methods indicates the reliability of the DEGs identified through RNA-Seq.

### Key genes related to muscle development

The contractile activity of the pectoral muscle has been found to be associated with myotube maturation and may serve as the stimulus for the expression of the neonatal MHC, as reported by Cerny and Starek [15]. During the period from E21 to E27, several genes related to muscle contraction, such as CSRP3, TNNT1, and TNNT3, were observed to be upregulated. The expression levels of these genes remained stable or continued increase at D0. Han et al. [16] demonstrated that CSRP3, predominantly expressed in skeletal muscle, plays a crucial role in promoting myoblast differentiation and maintaining the structure and function of normal muscle. Silencing CSRP3 has been found to result in a reduction in TNNT1 expression and consequent muscle atrophy, as reported by Cui et al. [17]. The importance of TNNT1 and TNNT3 in the contraction of striated muscles has been established by Ouyang et al. [1]. Deficiency of slow skeletal muscle TNNT1 has been linked to atrophy of type I slow fibers, as demonstrated by Wei et al. [18]. Additionally, downregulation of cell cycle genes, including Cyclin A2 (CCNA2), was observed from E21 to E27. CCNA2 is a crucial regulatory component of cyclin-dependent kinase 2 (Cdk2), which plays a vital role in promoting myoblast proliferation, as reported by Wicik et al. [19] and Yuan et al. [20]. The nterdependence between myoblast early differentiation and cell cycle activity has been documented, with the onset of irreversible cell cycle withdrawal occurring early during the differentiation process and being essential for the induction of the contractile phenotype [21]. It is plausible that the synchronized regulation of cell cycle genes and muscle contraction genes during myogenic differentiation serves as a means for the organism to regulate the accumulation of muscle mass during embryonic development.

Research on embryonic muscle development has shown that pectoral muscles undergo atrophy in the final days of incubation, without experiencing muscle fiber hypertrophy [22–24]. Several genes, such as TMEM182, DCN, and RPS6KA1, have been identified as being involved in muscle hypertrophy and are active between E21 and E27. TMEM182, a transmembrane protein, has been found to negatively regulate myogenic differentiation and fusion. Knocking out TMEM182 in mice leads to significant increases in body weight, muscle mass, muscle fiber number, and muscle fiber diameter [25]. DCN, a small dermatan sulfate proteoglycan, plays a role in normal muscle differentiation by suppressing MSTN activity during embryonic myogenesis [26–28].

Decorin (DCN) can induce the expression of MyoD and MyoG, promoting myoblast hypertrophy [29]. The differentiation of Dcn-null myoblasts into myotubes has been found to be delayed [30]. Furthermore, increased DCN expression in pectoral muscle during the late embryonic stage is associated with muscle weakness in low-score-normal (LSN) individuals [31, 32]. Posttranslational modifications mediated by 90 kDa ribosomal protein S6 kinase (RPS6KA1) are necessary for muscle differentiation and hypertrophy [33, 34]. S6K deficiency may lead to reduced protein synthesis and specifically trigger muscle cell atrophy in skeletal muscle cells [35]. The occurrence of skeletal muscle hypertrophy is attributed to higher rates of protein synthesis compared to protein degradation, as proposed by Sun et al. [36]. The spatial and temporal expression patterns of these genes align with the manifestation of muscle hypertrophy, highlighting the crucial role of these differentially expressed genes in the early stages of myogenesis.

Skeletal muscle development during embryogenesis requires precise regulation of myoblast adhesion and fusion. Myoblast fusion occurs after muscle specification and early differentiation, which are regulated by the myogenic regulatory factors (MRFs) MYF5, MYOD, and MYOG. MyoD activation is crucial for determining terminal differentiation, while MyoG is necessary for embryonic myoblast differentiation and fusion during hypertrophy [37, 38]. After E27, specific muscle fusion genes, such as myogenin (MyoG), transmembrane protein 8C (TMEM8C) [39], and disintegrin and metalloproteinase 12 (ADAM12) was observed to be downregulated. ADAM12 protein levels decrease in undifferentiated myoblasts but transiently increase during the onset of differentiation when myoblasts merge into multinucleated myotubes [40, 41]. Additionally, TMEM8C (myomaker), a muscle-specific membrane protein that plays a crucial role in initiating myoblast fusion and the formation of hemifusion intermediates [42–44].Its expression is regulated by MyoD and MyoG, both of which are early differentiation markers expressed in proliferating muscle cells [45].

The findings suggest that the coexistence of multiple myoblast fusion genes within muscle masses, exerting both temporal and spatial regulation over the fusion apparatus. Notably, the differentially expressed genes identified between E21 and D0 may modulate myoblast proliferation and early differentiation, cell cycle withdrawal, myoblast fusion, and myofiber formation in a temporal and spatial manner.

### Key genes related to muscle energy metabolism

The glycogen metabolism of the pectoral muscle plays a crucial role in providing glucose during pipping and hatching [46]. In late-term embryonic development in poultry, the pectoral muscle acts as a glucogenic energy store to be used when needed in preparation for hatching [47]. Activation of AMPK can enhance various processes, including increased fatty acid oxidation [48], glucose uptake, and glycogenesis, while reducing gluconeogenesis [49–52]. The AMPK signaling pathway involves genes such as phosphofructokinase-1 (PFKM) and adiponectin (ADIPOQ), both of which show increased expression from E21 to hatching.

Adiponectin (ADIPOQ) is a molecule secreted by adipocytes that promotes fatty acid oxidation, glucose uptake, and inhibits gluconeogenesis [53]. In muscle, ADIPOQ activates AMPK phosphorylation and activity, which is necessary for its effects on fatty acid oxidation and glucose transport. The expression of ADIPOQ after E21 suggests that it facilitates the utilization of fatty acids in muscle through the AMPK pathway, ensuring glucose and lipid homeostasis in the embryo. Moreover, the expression of other genes involved in the AMPK signaling pathway, such as GYS1, PYGM, PRKAA2, and ACACB, also increases after E27. According to O'Neill et al. [54], the phosphorylation of ACACB by AMPK is essential for the oxidation of fatty acids in skeletal muscle. The enzyme GYS1 regulates the rate of muscle glycogen synthesis and increases the activation of AMPK [55]. Additionally, PRKAA2 encodes a catalytic subunit of AMPK that positively regulates glycolysis [56]. The study's findings suggest that glucose is utilized by the pectoral muscle during pipping to support its activity and glycogen accumulation in the muscle is crucial during the later stages of incubation.

The development of skeletal muscle is a highly regulated process that involves the orchestration of multiple genes, transcription factors, noncoding RNAs, and signaling pathways [57, 58]. Among these, miRNAs serve as critical posttranscriptional regulators that fine-tune gene expression dynamics [59]. However, there is a lack of comprehensive studies examining the dynamics of miRNAs during duck muscle development. Previous studies by Gu et al. [7]and Li et al. [8, 9] have explored miRNA expression profiles during specific embryonic days of duck breast muscle development. Yet, a comprehensive study on the dynamics of miRNA expression during muscle development in black Muscovy ducks is still needed, with a focus on identifying key properties of miRNAs. To address this gap, this study examined the expression patterns of miRNAs in black Muscovy duck breast muscle across embryonic and posthatching periods. Seventy differentially expressed miRNAs (DEMs), including 55 known and 15 novel miRNAs, were identified through pairwise comparisons of libraries across four developmental stages. The observed miRNA expression patterns showed greater regional differences during early developmental stages (e.g., E21 vs. E27 and E27 vs. D0) than during late stages (e.g., D0 vs. D7). These findings suggest that the prehatching period may play a crucial role in the development of Muscovy duck muscle.

To gain a better understanding of the mechanism underlying the regulation of muscle development by miRNAs and their targets, a network of interactions between miRNAs and mRNAs associated with muscle development was established. Within this network, the musculoskeletal embryonic nuclear protein 1 (MUSTN1) gene was identified as a core gene and found to be targeted by three miRNAs: miR-206, miR-1a-3p, and miR-130c-5p. Of these, miR-206 and miR-1a-3p are muscle-specific miRNAs that have a significant influence on various muscle differentiation processes [60, 61].

The expression of the MUSTN1 gene significantly increased from E21 to E27 (*P* < 0.05), and the elevation persisted until postnatal day 7. The MUSTN1 gene plays a role in regulating skeletal muscle hypertrophy and myofusion, and it has been linked to the rapid development of the pectoral muscle in ducks [62–64]. The heightened expression of the MUSTN1 gene during the late embryonic phase suggests its crucial involvement in myofusion before hatching (D0) and the positive regulation of muscle growth during postnatal stages. Notably, our target validation data revealed that let-7b targets IGF2BP1 and MEF2C.

Let-7b has been found to have exert dual effects on skeletal muscle growth, acting as a regulator of the growth hormone receptor (GHR) and inhibiting insulin-like growth factor 2 mRNA-binding protein 3 (IGF2BP3) [65, 66]. The IGF2BP1 gene has been extensively studied in animal muscle development and is considered a prominent candidate gene associated with body weight and breast muscle weight in chickens and ducks [67–70]. Based on this, we propose that let-7b may regulate duck breast muscle development by targeting specific sequences in IGF2BP1 and myocyte enhancer factor 2C (MEF2C).

This study confirms that miR-301a-3p is the top downregulated miRNA from E21 to E27 and that it suppresses the expression of ANKRD1. Cardiac ankyrin repeat protein had been identified as a muscle atrophy marker [71]. ANKRD1 plays a crucial role in biological processes related to muscular growth, such as skeletal muscle tissue development and muscle cell differentiation linked to myogenesis [72, 73]. Given these functions, ANKRD1 may be critical for successful muscle hypertrophy. Another study has found that miR-301b-3p inhibits Rb1cc1 expression to control myogenic differentiation in chicken primary myoblasts [74]. Based on the available evidence, it can be concluded that the miR-301a-3p/ANKRD1 pathway has the potential to regulate muscle development.

## Conclusions

This study involved full-length transcriptome profiling and deep miRNA sequencing in the skeletal muscle of black Muscovy ducks at four different stages of development. The research uncovered unique miRNA expression profiles for each developmental stage, providing new insight into the regulatory network of muscle development. The study also identified several miRNAs and genes that may work together to regulate secondary myofiber differentiation during embryonic stages and muscle hypertrophy during postnatal stages. The study found that miR-301a-3p plays a role in promoting the proliferation and differentiation of satellite cells by targeting Ankrd1. These results can improve understanding of the regulatory functions of miRNA networks and shed light on the molecular mechanisms involved in breast muscle development. Furthermore, the findings may have practical implications for duck breeding.

## Methods

### Ethics statement

The animal model and experimental procedures used in this experiment were approved by the Animal Ethics Committee of the Institute of Animal Husbandry and Veterinary, Jiangxi Academy of Agricultural Science (JXAAS 2020–0025). The experiments were performed according to the Regulations for the Administration of Affairs Concerning Experimental Animals and the Standards for the Administration of Experimental Practices, as well as the ARRIVE guidelines version 2.0.

### Sample collection and RNA preparation

Black Muscovy duck eggs were obtained from a commercial farm (Nanchang City, Jiangxi, China). All eggs used in this study were incubated in one incubator with the same conditions at the same time, and black Muscovy ducks were raised at a commercial farm (NanChang City, Jiangxi, China) under the same conditions and were fed the same diet. The black Muscovy ducks used for sample collection at D0 were not fed. Three black Muscovy ducks per time point were randomly selected and sampled on Days 21 (E21) and Day 27 (E27) of the incubation period, on Hatching Day (D0) and on Day 7 (D7) post-hatching. In accordance with animal welfare guidelines, we conducted humane euthanasia on the ducks by inhaling carbon dioxide and cervical dislocation after fasting for about 12 h. Breast muscle tissues of the 3 individuals at time point were rapidly dissected, immediately snap-frozen in liquid nitrogen and then stored at -80 °C until RNA extraction.

### RNA isolation

Individual extraction of pectoral muscle RNA was performed using TRIzol reagent (Invitrogen, CA, USA) in accordance with the manufacturer's guidelines, with 10 μg of tissue being utilized for each sample. Monitoring of RNA degradation and contamination was carried out by subjecting the samples to 1% agarose gel electrophoresis. The purity of the RNA was determined through analysis using a NanoPhotometer® spectrophotometer (IMPLEN, CA, USA). Measurement of RNA concentration was accomplished using the Qubit® RNA Assay Kit within the Qubit® 2.0 Fluorometer (Life Technologies, CA, USA). To eliminate genomic DNA, each sample was treated with RNase-free DNase I at room temperature for 15 min and eluted with 50 μl of RNase-free water from Invitrogen. Lastly, RNA integrity was evaluated using the RNA Nano 6000 Assay Kit of the Agilent Bioanalyzer 2100 System (Agilent Technologies, CA, USA).

### Library construction and SMRT sequencing

Subsequently, the RNA samples were combined into a single sample with equal amounts for the construction of the Iso-Seq library. The Iso-Seq library preparation followed the isoform sequencing protocol (Iso-Seq) using the Clontech SMARTer PCR cDNA Synthesis Kit and the BluePippin Size Selection System Protocol, as described by Pacific Biosciences (PN 100–092-800–03). For transcriptome sequencing and small RNA library creation, 3 μg of total RNA per sample was used as input material. The NEBNext® Multiplex Small RNA Library Prep Set for Illumina® (NEB, USA) was utilized to generate sequencing libraries, following the manufacturer's recommendations, with index codes added to assign sequences to each sample. Sequencing was conducted on a HiSeq 2500 instrument. Novogene Co., Ltd. (Tianjin, China) performed all sequencing procedures. The raw data underwent standard processing methods, resulting in the acquisition and annotation of full-length transcriptome and microRNA sequences.

### Full-length sequencing and analysis pipeline

We performed initial data processing in accordance with the Iso-Seq standard pipeline, combining all raw data. Using SMRTlink (version 5.1) software, we generated the circular consensus sequence (CCS) from the initial data. Based on the 5' and 3' adapters, as well as the poly(A) tail, the CCS was then divided into two categories: full-length and non-full-length reads. Full-length reads contained both the 5' and 3' primers, along with a poly(A) tail signal preceding the 3' primer. To identify transcript clusters, we used Iterative Clustering for Error Correction (ICE), which involved pairwise alignment and reiterative assignment of full-length reads. In order to obtain high-quality isoforms, we polished the cluster consensus reads using non-full-length reads and the Arrow software. We corrected any additional nucleotide errors in the consensus reads using LoRDEC software, leveraging the Illumina RNA-seq data [75]. Finally, to ensure non-redundancy in our corrected consensus reads, we employed CD-Hit-Est25 and obtained the final transcripts for subsequent analysis.

### Differential expression analysis

Reference sequences were obtained from the Pacific Biosciences RS sequencing instrument in full-length transcripts. Mapping of HiSeq sequencing reads to the reference sequences was performed using Bowtie, and statistical analysis of read count was conducted on the mapping results using RSEM. Differential expression analysis between two stages was carried out utilizing the DESeq R package (1.10.1). DESeq employs statistical algorithms based on the negative binomial distribution to determine differential expression in digital gene expression data. To control the false discovery rate, the resulting *P* values were adjusted through Benjamini and Hochberg’s approach. Genes with a *P* < 0.05, as determined by DESeq, were annotated as differentially expressed.

For microRNA expression analysis, the TPM (transcripts per million) method was used. The following normalization formula was employed: normalized expression = mapped read count / total reads × 1,000,000. Similar to the gene expression analysis, differential expression analysis between two stages was carried out using the DESeq R package (1.8.3). The *P* values were adjusted using the Benjamini and Hochberg method. Significantly differential expression was defined as a *P* value of less than 0.05 by default.

### Integrated analysis of differentially expressed genes (DEGs) and differentially expressed miRNAs (DEMs)

The miRanda software was utilized to predict target genes in order to understand the biological function of miRNAs [76]. It is well-known that miRNAs typically decrease the expression of their target genes. Therefore, differentially expressed target genes that displayed a negative correlation with the corresponding miRNAs in the same comparison group were carefully selected for further investigation. To gain a better understanding of the functional roles of these target genes, a GO enrichment analysis was performed using the Wallenius noncentral hypergeometric distribution. This analysis was conducted by employing GOSeq, which is a widely-used tool for gene ontology analysis [77]. Furthermore, the predicted target genes were also annotated using the KEGG database, which is a comprehensive resource for understanding molecular interactions and associated pathways [78]. To identify the major signal transduction pathways influenced by the target genes, KEGG pathway analysis was carried out. This analysis revealed the key pathways that these genes are involved in, shedding light on the potential molecular mechanisms underlying the observed changes. Notably, the statistical enrichment of the target genes in the KEGG pathways was investigated through the use of KOBAS (v2.0), a software package designed specifically for this purpose. This statistical enrichment analysis provided valuable insights into the significance of the target genes within the context of the identified KEGG pathways [79, 80].

### miRNAs‒mRNAs interaction analysis

The expression data of mRNAs and miRNAs were integrated using the MAGIA web tool In order to anticipate miRNA targets and examine the connection between miRNAs and mRNAs, we integrated the expression data of mRNAs and miRNAs via the MAGIA web tool [80] and demonstrated it with Cytoscape [81]. For the visualization in Cytoscape, we only considered the 250 most significant interactions. We selected networks with considerable enrichment (> 30) which are associated with cell development or muscle growth. These networks were merged to display the interaction between the DEGs and DEMs.

### Validation of sequencing results by qRT‒PCR

For quantitative determination of the reliability of the sequencing data, qRT‒PCR was carried out to test the expression levels of 16 DEGs and 10 DEMs, which were randomly selected. Reverse transcription of mRNA to cDNA was performed using the RevertAid First Strand cDNA Synthesis Kit (K1622; Thermo Scientific) according to the manufacturer’s instructions. The relative mRNA expression level was determined using GAPDH as a housekeeping gene in each sample. Both forward and reverse primers are listed in Supplemental Table S1. U6 was chosen as the internal control for miRNA. Reverse transcription-PCR was used to synthesize cDNA using a miRNA 1st Strand cDNA Synthesis Kit (by stem‒loop) (Vazyme, Nanjing, China). Primers were obtained based on the mature sequences of the miRNAs identified in the present study (Supplemental Table S2). Mature sequences were obtained in the miRBase database, according to each miRNA's name. A CFX96TM real-time system (Bio-Rad, Hercules, CA, USA) was used to perform qRT-PCR with a SYBR PrimerScriptTM real-time PCR kit (TaKaRa, Dalian, China). The 2 − ΔΔCt method was used to measure the levels of relative expression, and expression differences were analysed by Student’s t test [82]. A *P* value of 0.05 was considered to indicate statistical significance.

### Cell culture and isolation

The breast muscles of black Muscovy ducks at Embryonic Day 27 (E27) were used to isolate satellite cells as described in previous reports [83]. Satellite cells were cultured with Dulbecco’s modified Eagle medium (DMEM) (HyClone, Logan, UT, USA) containing 10% foetal bovine serum (CLARK, Worcester, MA, USA) at 37 ℃ in a humidified atmosphere with 5% CO_2_.

### Dual luciferase reporter assay

The miR-301a-3p inhibitor, negative inhibitor, miR-301a-3p mimic, and negative mimic were synthesized by GenePharma (GenePharma, Shanghai, China) (Supplemental Table S3). The Muscovy duck ANKRD1 3’-UTR comprising the expected complementary site of miR-301a-3p (wild type) and its identical sequence with the mutant sequences of specific complementary sites of miR-301a-3p (mutant) were inserted into the pmirGLO luciferase vector (Promega, Madison, WI, USA) (Supplemental Table S4). Satellite cells were cotransfected with pmirGlo-ANKRD1 3’-UTR wild type or pmirGLO-ANKRD1 3’-UTR mutant, and miR-301a-3p mimics or mimic-NC was cotransfected into the cells. After transfection for 48 h, the luciferase activity was measured using a Dual-Luciferase Reporter Assay System (Promega, Madison, WI, United States). The luminescence activity of firefly luciferase and Renilla was also detected using a multifunctional microplate reader (BioTek, Winooski, VT, USA) following the manufacturer’s instructions.

### Cell proliferation assays

Cell proliferation was evaluated using a Cell Counting Kit-8 (CCK-8; Meilunbio, Dalian, China) in accordance with the manufacturer ‘s instructions. Satellite cells were seeded in 96-well plates and transfected with miR-301a-3p mimics or inhibitors. The cells were transfected for 12, 24, 36 and 48 h, and then, according to the instructions of the manufacturer, 10 µl of CCK-8 reagent was added to each well. The cells were incubated for 1 h at 37 °C. Absorbance was measured at 450 nm using a Thermo ScientificTM Varioskan LUX (San Jose, CA, USA).

### Induction of myogenic differentiation of satellite cells

Primary myosatellite cells were treated with myogenic differentiation medium (2% horse serum, 1% double antibodies, 1% GlutaMAX, 90% high-glucose DMEM) for 0, 2, 4, and 6 days, and the expression levels of miR301a-3p, ANKRD1 and the myoblast differentiation genes MyHC, MyoD, MyoG and Myf5 were measured by qRT‒PCR.

### Supplementary Information

**Supplementary Material 1.**

**Supplementary Material 2.**

**Supplementary Material 3.**

**Supplementary Material 4.**

**Supplementary Material 5.**

**Supplementary Material 6.**

**Supplementary Material 7.**

**Supplementary Material 8.**

**Supplementary Material 9.**

**Supplementary Material 10.**

**Supplementary Material 11.**

**Supplementary Material 12.**

**Supplementary Material 13.**

## Data Availability

The raw sequence data reported in this paper have been deposited in the the Genome Sequence Archive in BIG Data Center, Beijing Institute of Genomics, Chinese Academy of Sciences (GSA: CRA011648), which are publicly accessible at https://ngdc.cncb.ac.cn/search/?dbId=gsa&q=CRA011648.

## Abbreviations

DEGs: Differentially expressed genes
DEMS: Differentially expressed miRNAs
CCS: Circular consensus sequence
SC: Satellite cell
CCNA2: Cyclin A2
Cdk2: Cyclin-dependent kinase 2
CSRP3: Cysteine and glycine-rich protein 3
TNNT1: Troponin T1
TNNT3: Troponin T3
DCN: Decorin
LSN: Low-score-normal
RPS6KA1: Ribosomal protein S6 kinase A1
ADAM12: ADAM metallopeptidase domain 12
MyoD: Myogenic differentiation protein
MyoG: Myogenin
TMEM8C: Transmembrane protein 8C
PFKM: Phosphofructokinase
ADIPOQ: Adiponectin
AMPK: AMP-activated protein kinase
GYS1: Glycogen synthase 1
PYGM: Muscle glycogen phosphorylase
PRKAA2: Protein kinase AMP-activated catalytic subunit alpha 2
ACACB: Acetyl-CoA carboxylase beta
MUSTN1: Musculoskeletal embryonic nuclear protein 1
ANKRD1: Ankyrin repeat domain 1
IGF2BP1: Insulin like growth factor 2 mRNA binding protein 1
MEF2C: Myocyte enhancer factor 2C
GHR: Growth hormone receptor
